# A one-size-fits-all approach to data-sharing will not suffice in lifecourse research: a grounded theory study of data-sharing from the perspective of participants in a 50-year-old lifecourse study about health and development

**DOI:** 10.1186/s12874-023-01940-6

**Published:** 2023-05-16

**Authors:** Jane Reeves, Gareth J. Treharne, Mihi Ratima, Reremoana Theodore, Will Edwards, Richie Poulton

**Affiliations:** 1 Department of Psychology, University of Otago, PO Box 56, Dunedin, Aotearoa, 9054 New Zealand; 2 Te Pou Tiringa, New Plymouth, Aotearoa, New Zealand; 3 Dunedin Multidisciplinary Health and Development Research Unit, Department of Psychology, University of Otago, PO Box 56, Dunedin, Aotearoa, 9054 New Zealand; 4 Taumata Associates, New Plymouth, Aotearoa, New Zealand

**Keywords:** Data-sharing, Longitudinal research, Lifecourse, Research participant perspectives, Indigenous peoples, Data sovereignty, Confidentiality, Anonymity, Research consent

## Abstract

**Background:**

Data-sharing is increasingly encouraged or required by funders and journals. Data-sharing is more complicated for lifecourse studies that rely upon ongoing participation, but little is known about perspectives on data-sharing among participants of such studies. The aim of this qualitative study was to explore perspectives on data-sharing of participants in a birth cohort study.

**Methods:**

Semi-structured interviews were conducted with 25 members of the Dunedin Multidisciplinary Health and Development Study when aged between 45 and 48 years. Interviews were led by the Director of the Dunedin Study and involved questions about different scenarios for data-sharing. The sample consisted of nine Dunedin Study members who are Māori (the Indigenous peoples of Aotearoa/New Zealand) and 16 who are non-Māori.

**Results:**

Principles of grounded theory were applied to develop a model of participant perspectives on data-sharing. The model consists of three factors that inform a core premise that a one-size-fits-all approach to data-sharing will not suffice in lifecourse research. Participants suggested that data-sharing decisions should depend on the cohort and might need to be declined if any one Dunedin Study member was opposed (factor 1). Participants also expressed a proven sense of trust in the researchers and raised concerns about loss of control once data have been shared (factor 2). Participants described a sense of balancing opportunities for public good against inappropriate uses of data, highlighting variability in perceived sensitivity of data, and thus a need to take this into account if sharing data (factor 3).

**Conclusions:**

Communal considerations within cohorts, loss of control over shared data, and concerns about inappropriate uses of shared data need to be addressed through detailed informed consent before data-sharing occurs for lifecourse studies, particularly where this has not been established from the start of the study. Data-sharing may have implications for the retention of participants in these studies and thus may impact on the value of long-term sources of knowledge about health and development. Researchers, ethics committees, journal editors, research funders, and government policymakers need to consider participants’ views when balancing the proposed benefits of data-sharing against the potential risks and concerns of participants in lifecourse research.

## Background

Data-sharing is broadly defined as the process of making anonymised individual-level raw data from research routinely available to other researchers and often the general public too [1]. In recent years there has been a radical transformation of data-sharing practices, and it is increasingly common to find raw data shared directly alongside academic articles on journal websites or via online repositories. These changes to data-sharing practices are driven by the potential benefits of having access to raw data [1]. For example, the sharing of raw data has been central to providing prompt solutions to emerging problems, and COVID-19 is a case in point [2]. However, data-sharing comes with many ethical considerations, and there is a pressing need for research to address the privacy implications for participants and the wider implications for participants’ ongoing engagement in research [3]. Data-sharing decisions become increasingly complex for studies that are not cross-sectional, and particular tensions are likely to exist for research participants who are taking part in ongoing lifecourse studies due to their ongoing participation [3].

Data-sharing is a core component of the ‘open science’ movement, which advocates for transparency in all components of scientific research in order to benefit from pooling and reusing data as well as being a means of overcoming concerns about findings that do not replicate in other studies and avoiding unethical practices such as fabrication of data [1]. Funders and journals have increasingly been implementing data-sharing policies including stipulations for data-sharing plans or mandatory inclusion of data for peer-review and publication. For example, the US National Institutes of Health has announced a requirement for all data from projects they fund to be made public from 2023 [4] with scope for variations in the extent of data-sharing due to extenuating reasons such as sensitivity of the data and/or potential privacy concerns of participants. Such policies are often interpreted as broad statements and run the risk of data being shared without careful planning specific to the research methodology in question. Moreover, the application of broad data-sharing policies can directly conflict with ethical guidelines and data policies that aim to protect the rights of research participants if privacy and informed consent are not addressed appropriately [3, 5]. There is a lack of consistency across data-sharing policies that means researcher have to handle aspects of different guidance; moreover, real-world decisions about the practice of data-sharing are in the hands of researchers as opposed to the participants in studies, and this is where complex ethical concerns can arise [6, 7].

At present, there are gaps in knowledge about the perspectives on data-sharing among different stakeholders. Of particular concern, research into data-sharing has rarely included the views of long-term research participants [3]. The limited existing research into the perspectives of research participants on data-sharing has focused on one-off studies in health, mostly specific to genomic or biobank research [8–10]. These studies have shown that research participants are cautiously open to individual-level data-sharing. However, openness towards data-sharing is partially determined by how informed consent is framed to participants, and participants tend to indicate a preference for there to be greater limitations on data-sharing when these options are made available and explained in comparison to when researchers ask for broad consent for data-sharing [10, 11].

Views about data-sharing have also been linked to research participants’ views on the sensitivity of the data in question, with data related to mental health conditions, sexual or reproductive health, and alcohol use considered more sensitive than some other types of data by participants [12]. The willingness of participants to share data has also been found to potentially be contingent on who is receiving the data and the potential stigma associated with the sensitivity of the data being shared [12–14]. Findings from a meta-analysis showed that willingness to share data is more likely among participants who have familiarity with genomic research as well as those with long-term experience in a particular healthcare setting, the presence of a heritable condition within the family, and trust in local and nationwide research infrastructures [15]. However, participants have been found to report a lower willingness to contribute data for genomic research when the data are to be shared with multiple researchers or agencies [15].

Past research has shown that research participants are aware of the potential for data-sharing to create public good but at the same time have concerns related to privacy, misuse of data, and discrimination on the basis of health conditions or ethnicity [8, 9, 16, 17]. Data-sharing is increasingly being applied within Western science policy frameworks [7]. Historically, in Aotearoa/New Zealand, research undertaken by non-Māori on Māori (the Indigenous people of Aotearoa/New Zealand) has provided little, if any, benefit for Māori [18]. The growing Indigenous data sovereignty movement seeks to uphold the rights of self-determination for Indigenous peoples to control and govern their Indigenous data to realise Indigenous aspirations [19, 20]. This includes accepting that data are subject to the laws of the country or territory where it is collected and stored [20, 21].

More widely, research participants have argued for increased transparency about the process of data-sharing, implementing tailored data-sharing plans which suit the needs of the communities involved in the research, and the necessity of maintaining some form of autonomy over data, for example in relation to secondary analyses [8, 9, 22–24]. Research participants have been treated as a homogenous group in the literature on data-sharing, with a general lack of consideration about participant diversity or changes in data-sharing policies and associated processes of informed consent. Participants in lifecourse studies are likely to have particular concerns about data-sharing because of the amount of data collected about them over an extended period of time, which can lead to an increasing risk of what is referred to as ‘re-identification’ of participants even when data have been anonymised before being shared [25]. Participants commonly have a commitment to further data being collected and a strong motivation based on creating public benefit through their contributions, but concerns about data-sharing are likely to impact the ongoing retention of participants, and this could act as a deterrent to participating in long-running studies [25].

### Aims

Based on the gaps in existing literature, there are pressing questions about the application of data-sharing protocols for lifecourse studies. Input from research participants themselves is needed to understand their concerns and what should be required to ensure appropriate informed consent about data-sharing in such studies. Addressing these questions is required in order to understand what participants see as the boundaries to data-sharing and the implications for their ongoing participation in research. The present qualitative study was conducted with selected participants from the Dunedin Multidisciplinary Health and Development Study (henceforth, ‘the Dunedin Study’), which is an ongoing lifecourse research project that began in 1972–1973 with N = 1,037 study members at foundation [26]. The Dunedin Study provides a highly relevant exemplar for building a theoretical understanding of the views of participants in lifecourse research regarding the appropriateness of various data-sharing processes for different forms of data gathered over 50 years.

The specific aims of the present qualitative study on data-sharing were:

1. To explore the perspectives on data-sharing of participants in the Dunedin Study as an exemplar of a long-running lifecourse study.
2. To build an understanding of participants’ interpretation of existing arguments for data-sharing in relation to their ongoing participation in lifecourse research.
3. To gain insight into the concerns that lifecourse research participants have about data-sharing.
4. To develop a novel theory of participants’ perspectives on whether aspects of data-sharing are appropriate for lifecourse research.

## Methods

A constructivist grounded theory approach [27] was used to design a process of engaging with a range of members of the ongoing Dunedin Study. Engagement was carried out separate to the standard periodic assessments within the Dunedin Study. The most recent assessment occurred when the Dunedin Study members were aged 45 years in 2017–2019. Grounded theory methodology seeks to explain the occurrence of a social process within a specific environment [28]. The application of grounded theory methodology is appropriate when there is a need to develop new theoretical models and fits the aim of the present study to explore lifecourse research participants’ perceptions of data-sharing [28, 29].

Ethics approval was granted by the Human Ethics Committee of the University of Otago (reference 18/075). The study was conducted in concordance with relevant regulations and ethics principles. All participants gave informed consent to participate in an interview after being invited by a member of the research staff of the Dunedin Study based on stratification characteristics on file. Purposive sampling of members of the Dunedin Study initially centred on efforts to include Māori and non-Māori participants. In addition, efforts were made to stratify by gender and education. Subsequently, a purposive effort was added to include some people with and without children across these other stratification characteristics, as per grounded theory methods in developing the sampling based on emerging issues of relevance [27].

The study was run by a Master’s student (the first author) who subsequently continued the research as a staff member. The other authors were supervisors/advisors of the Master’s project and all met regularly during the research to reflect on the progress and discuss roles and responsibilities, including the power dynamics among the researchers and in relation to the interviewing of participants. One-on-one interviews were conducted in order to elicit individual perspectives from Dunedin Study members on data-sharing whilst also maintaining confidentiality. A semi-structured approach was adopted and provided flexibility in the interaction between the participant and the interviewer [30]. As semi-structured interviews rely on rapport between the participant and the interviewer [31], the Director of the Dunedin Study (the last author) conducted the interviews. The interviewer has wide knowledge of the Dunedin Study and had established relationships with the participants that had been built up over almost 40 years having first interviewed participants when they were aged 13 in 1985–1986. The familiarity of the interviewer was felt to be more important for rapport and a sense of confidentiality for participants over any concern about power between the interviewer and participants, who were frank in sharing their perspectives. A schedule of open-ended questions was designed with input from all authors to elicit information about data-sharing and to encourage participants to discuss what they perceived as important in relation to data-sharing [32, 33].

The constructivist approach to grounded theory involved iteratively analysing the data using constant comparison at the same time as collecting further informative data in order to produce a substantive novel theory ‘grounded’ in participants’ perspectives on the emergent constructs related to data-sharing [27]. Within this approach, the theorisation of the central phenomena of interest is co-constructed through the researchers’ interpretation of the participants’ experiences [27]. In the present study, the first author led the analyses with input from the principal investigator of this specific project (the second author) as well as the interviewer (the last author) and the three other authors (who are Māori researchers). Power dynamics were regularly discussed in group supervision meetings and during individual discussions. No conflicts of interest were experienced in relation to roles in this project.

### Demographics of interview participants

Demographic information was confirmed through a self-report questionnaire prior to each interview. Fourteen women and 11 men participated in this qualitative study, and all participants were cisgender (non-transgender). The age range was 45–48 years old. One participant did not state their sexuality and all others listed their sexuality as straight. The cross-tabulation of demographic characteristics is provided in Table 1. Nine participants listed their ethnicity as Māori (seven of whom also listed Pākehā/New Zealand European ethnicity); 15 as Pākehā/New Zealand European; and one as New Zealand Asian ethnicity (specific details excluded to maintain confidentiality). Five participants had no children, and twenty participants had between two and five children (median three).

**Table 1:** Stratification details of the sample of Dunedin Study members

Ethnicity	Gender	Education	Any children	n in this stratum
Māori	Female (cisgender)	University education	Children	4
Māori	Female (cisgender)	University education	No children	0
Māori	Female (cisgender)	No university education	Children	2
Māori	Female (cisgender)	No university education	No children	0
Māori	Male (cisgender)	University education	Children	1
Māori	Male (cisgender)	University education	No children	0
Māori	Male (cisgender)	No university education	Children	2
Māori	Male (cisgender)	No university education	No children	0
Non-Māori	Female (cisgender)	University education	Children	2
Non-Māori	Female (cisgender)	University education	No children	2
Non-Māori	Female (cisgender)	No university education	Children	4
Non-Māori	Female (cisgender)	No university education	No children	0
Non-Māori	Male (cisgender)	University education	Children	3
Non-Māori	Male (cisgender)	University education	No children	2
Non-Māori	Male (cisgender)	No university education	Children	2
Non-Māori	Male (cisgender)	No university education	No children	1

### Interview procedure

Potential participants were invited to take part in an interview. Invitations were made either through a secure email address or in person during scheduled assessments at the Dunedin Study unit location. The main reason given for not participating was being too busy. Individuals who attended an interview received an information sheet in advance and were able to ask questions before the start of the interview. All agreed to go ahead with the interview and signed consent forms before the start of the interview. Three of the 25 participants were interviewed via telephone or videoconferencing, and they returned signed consent forms by post and also confirmed consent verbally before the interview began.

Interviews were conducted between July 2018 and December 2020 and involved a semi-structured question guide. The questions covered various aspects of and motivations for data-sharing and sought views on hypothetical data-sharing situations that might be applied to the Dunedin Study in the future (e.g., raw data being published alongside journal articles). The audio recordings of the interviews were transcribed verbatim and checked for accuracy before analysis.

### Applying reflexivity

Reflexivity was applied throughout the project to interrogate our own perspectives of data-sharing, in order to identify and collectively consider how our values and experiences informed the co-construction of our emergent grounded theory [31]. The first author (JR) is a cisgender female Pākehā (non-Māori) researcher who initially led the analysis for her Master’s in Psychology and in an ongoing role as an assistant research fellow. The second author (GT) is a cisgender male Pākehā researcher who has expertise in longitudinal health research, qualitative methods, and research ethics. He was the lead supervisor of the first author and co-ordinated the finalisation of the grounded theory analysis. The third author (MR) is a cisgender female Māori researcher with expertise in Māori health research, health promotion, and research ethics. The fourth author (RT) is a cisgender female Māori researcher who is the co-director of a national collaborative research centre and has expertise in longitudinal research and Māori health research. The fifth author (WE) is a cisgender male Māori researcher who is a director of a Māori community-based research organisation and active in national research organisations and Māori community leadership. He has expertise in epistemology and Māori perspectives on research data and community development. The last author (RP) is a cisgender male Pākehā researcher who conducted all interviews based on his established relationship with Dunedin Study members. He is the Director of the Dunedin Study and a clinical psychologist. He has expertise in lifecourse research and application of research in policy and practice and was also a governmental Chief Science Advisor at the time of the interviews. The authors met in person and via teleconference at key junctures in the research process to discuss our collective reflections on the aims and emerging findings, and the two lead analysts met weekly during active periods of analysis to reflect on new data and constant comparison.

### Data analysis

The data analysis focused on the production of a novel theory of data-sharing using an iterative combination of inductive processes (drawing from participants’ narratives) and abductive processes (explaining expected and unexpected elements of the data) [27]. Existing frameworks for constructivist grounded theory were used to guide the analysis, particularly in the application of three levels of coding which correspond to increasingly higher levels of theoretical abstraction [34–36].

The initial open coding involved the transcripts being read and reread before being coded, with equal attention being paid to each component of text and comparison to other coded sections [35, 36]. Once all the interviews had been coded, the codes were listed, compared, and collapsed against each other to form the basis of tentative categories. Intermediate or selective coding involved amalgamating codes around a central construct, while theorising about connections between and within categories and whether the data were supportive of these interactions. Each interview was summarised as a ‘memo’, focusing on the analytical abstraction of two or three key ideas. Memos were shared between the two lead analysts to check for consistency and provide feedback on other points arising within the data, before being shared with the wider research team. Ongoing discussions were held among all authors, including consideration of views expressed by Māori and non-Māori participants. After iterative analysis of all ongoing interviews, the newer participants were found to echo similar conceptualisations of data-sharing, and therefore data saturation was confirmed after 25 interviews.

Advanced coding and theoretical integration involved making sense of the data and finalising the categories [35]. The tentative categories were tabulated for each participant by reviewing their transcripts again. At this stage, the third author analysed the nine Māori transcripts and the categories were discussed to ensure considerations for Māori participants were accounted for through a Māori lens. Following these discussions, the categories were expressed as factors within the theoretical model and reviewed by all authors.

## Results

**Fig. 1:**
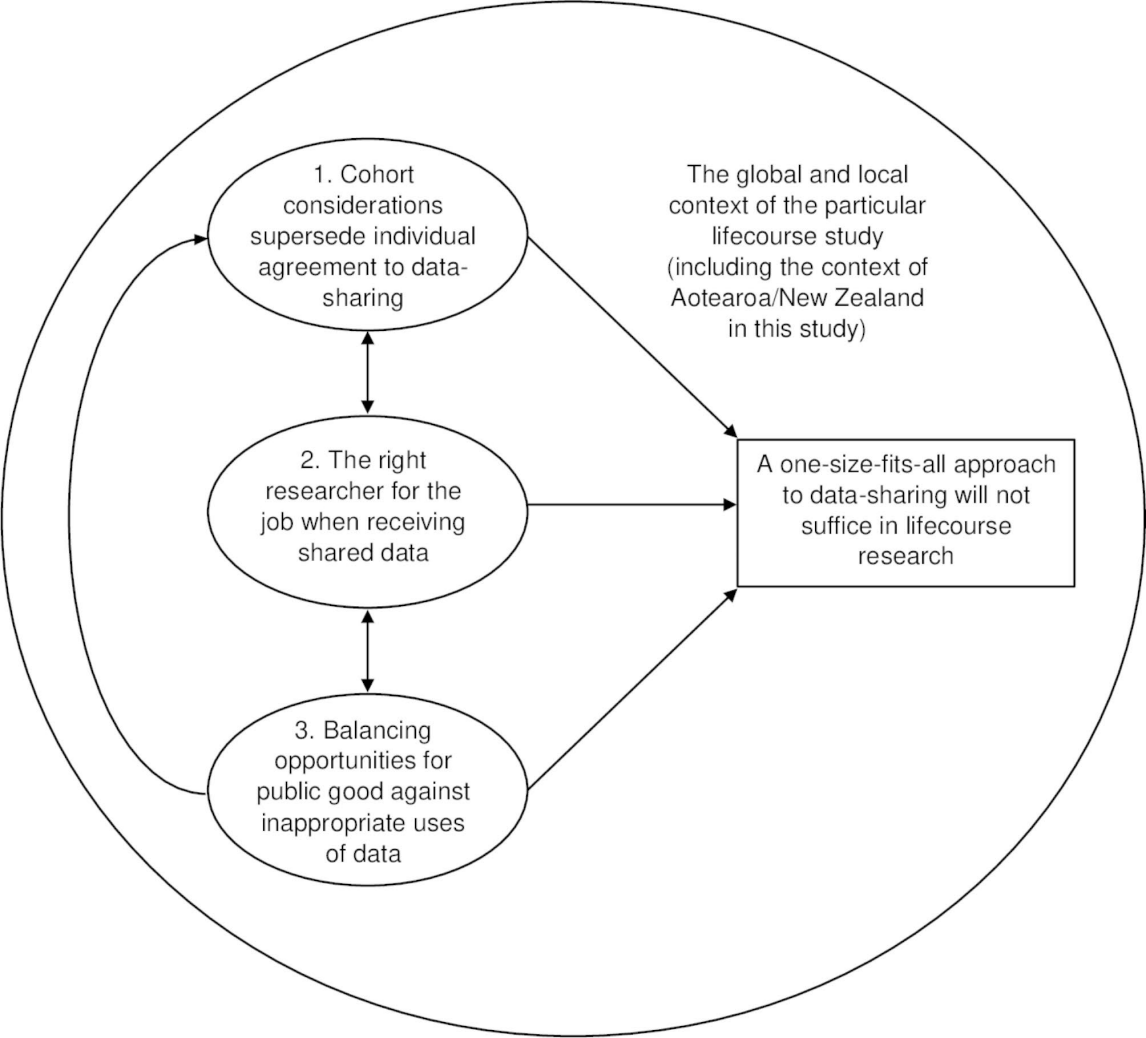
Visual representation of the core premise and related factors within the grounded theory model of participants’ perspectives on data-sharing in long-term lifecourse research

The grounded theory analysis led to the construction of a theoretical model about data-sharing from the perspective of lifecourse research participants, as depicted in Fig. 1. The overall model sits within the local context of Aotearoa/New Zealand amidst the global data-sharing debate. At the core of the model lies the premise expressed by participants that ‘a one-size-fits-all approach to data-sharing will not suffice in lifecourse research’ and relates to the interaction between three core factors: (1) ‘cohort considerations supersede individual agreement to data-sharing’; (2) ‘the right researcher for the job when receiving shared data’; and (3) ‘balancing opportunities for public good against inappropriate uses of data’. Figure 1 displays how the three factors feed into the core premise that data-sharing is more complicated than assumed and that a one-size-fits-all approach is unlikely to work in lifecourse research. Factor one on cohort considerations superseding individual agreement to data-sharing is positioned at the top to indicate its primacy in the theory, reflecting its primacy for participants. Arrows within the figure indicate the flow of impact evident across participants’ narratives. Specifically, having the right researcher for the job when receiving shared data impacts reciprocally with participant perspectives on cohort considerations and balancing opportunities for public good against inappropriate uses of data. Cohort considerations are also impacted unidirectionally by balancing opportunities for public good against inappropriate uses of data, which shape these cohort considerations. Participants are referred to by the interview number throughout the results section because any selected pseudonym would likely be the name of one of the approximately 1,000 participants in the ongoing overall Dunedin Study.

### Core premise: a one-size-fits-all approach to data-sharing will not suffice in lifecourse research

For many of the participants in this study, the interview was the first time they had considered data-sharing and the processes that would be appropriate for the data-sharing of lifecourse research data. Participants drew on their knowledge of the Dunedin Study and came up with comparable scenarios outside the study (e.g., taking part in other research) in order to make sense of how data-sharing aligns with research processes including funding applications, managing data-sharing requests from other researchers or agencies, and the storage, analysis and dissemination of research data. This led participants to reflect on how their views about data-sharing are influenced by the deep trust that participants have in the research team and the protocols applied in the wider study because the researchers have proven themselves to be trustworthy over the long duration of the study:

“I have complete, one hundred percent faith that that data is, not that I knew what happened to the data (laughs), blind faith that data was in safe hands and being managed.” – P12

In speaking about the degree of trust placed in the research team, participants highlighted that the positions of participants and researchers are different in lifecourse research compared to one-off studies because of the longer timeframes and regular interactions that enable trust to develop. They noted how successful lifecourse research requires participants and the research team to develop a mutually beneficial relationship, which is sustained by the level of trust that is developed. Participants attributed their willingness to honestly disclose whatever information was requested directly to the high quality of the relationships with the research team, and in turn felt that their honesty played a key role in data quality:

“I really value the model that you use and I think it’s a really high trust model, and the relationships that the people, as a [Dunedin] Study member, the people that we deal, with those trusting relationships. I personally have implicit trust when I come here” – P25

Participants spoke to the ‘culture’ of the research team which is one within which the interests of study members are protected and prioritised at every turn, and this contributed to the high level of trust placed in the team to appropriately safeguard data. From this perspective, the research team were described as ‘guardians’ of the data held on participants. Asking participants their perspectives on data-sharing reinforced some participants’ belief in the shared ethos of guardianship within the research team:

“the values that you’ve brought and predecessors and the whole team bring is the right values to look after us” – P6

Invariably, as interviews progressed, participants moved beyond a dichotomous yes/no answer to questions about hypothetical data-sharing scenarios. They described conditions where data-sharing would or would not be acceptable. These responses are described as three distinct factors that are inter-related, as shown in Fig. 1.

### Factor 1: cohort considerations supersede individual agreement to data-sharing

A consistent sentiment expressed by participants was that they would be open to researchers sharing their life-long research data only if their anonymity was guaranteed and only if all other participants were also in agreement about data being shared. Where participants were open to their own data being shared, a common rationale was that they had nothing particularly sensitive within their individual dataset:

“While I think I’m the average person, I may not be the average person. People’s sensitivity issues might be sensitive to all manner of things.” – P22

It was generally agreed that the sensitivity of data should also be factored in when determining whether the researchers should share research data. However, there was no overall consensus on what constitutes ‘sensitive’ data. For example, some participants constructed data as sensitive if the information related to crime, participants’ relationships, or mental health. In contrast, other general medical or health information was seen as less sensitive data and therefore more appropriate for sharing:

“all the medical side of it and everything like that, would be fine, but some people may not like travelling down the path as an individual and exposing themselves to mental health issues, with drugs or alcohol related things” – P7

Several Māori participants indicated that data of cultural significance should be treated as sensitive in various scenarios:

“my daughter put it up on Facebook [image of grandchild engaged in cultural practice] and coz it’s Māori and […] I just didn’t think it needed to be there” – P3

Comments also indicated that biological data has cultural significance and is sensitive from a Māori perspective:

“with Māoridom anything our hair, our fingernails, everything like that have a special part” – P8

Participants also articulated that it is particularly important that determinations about data-sharing should account for the “lowest common denominator” (P6) such that data-sharing decisions should be based on protecting any members of the cohort whose data could potentially generate harmful outcomes for that person if shared. The construction of a united community of participants led to the sentiment that data-sharing would only be appropriate if there was unanimous agreement across the cohort:

“if there came to be a decision and even just one person was uncomfortable, then I’d be like, cool, then none of us are doing it” – P20

### Factor 2: the right researcher for the job when receiving shared data

Participants shared insights into who outside of the research team should be granted access to the wider cohort data and expressed how it has to be the right researcher for the task required of the research and drew on metaphors of inappropriate people tackling jobs that they are not qualified to have. Participants explained that it felt inappropriate for funders or journal editors to make the ultimate decision about data being shared and were concerned that the hard work of the research team would be undermined by others having access to the data for no effort:

“I think on a personal level, well, I’m kind of happy with [data-sharing], so long as it’s not traced back to me personally or anything like that. I think it’s more that actually this study, unless the study as a group are involved in whatever else that’s going to be used, then perhaps not, because then that’s a lot of work for you guys to have done for somebody else to take all that data” – P20

When describing ideal external candidates with whom data may be shared, participants highlighted that a similar research ethos was crucial. They expanded on what constituted this ethos in these discussions, noting that external researchers’ values should align with the prioritisation of the research participants, and an opposition to financial gain being the motive for data-sharing or any aspect of research:

“I didn’t want it going into corporate hands where they could use it to make money” – P5

Some of the Māori participants noted that their trust in the research team was grounded in their trustworthy actions over time, which contrasted with experiences with other agencies and led them to see the Dunedin Study as a safe place to disclose information not only about themselves but also about their family. Concerns were raised about the possibility that data related to both Māori participants and the wider cohort may be misinterpreted or misused if shared with the wrong external agencies, particularly those who were outside of Aotearoa/New Zealand:

“so for it to be tika [true] and them to get the correct answers I believe they [external researchers] need to come back here rather than having the data themselves.” – P8

In the context of sharing data for replication of analyses, participants generally acknowledged the utility of this approach. Discussions tended to turn back to concerns about the research team losing control over the dataset. The loss of control was linked to the misuse or misinterpretation of data by external researchers, as there was no guarantee that they shared the same values in relation to research ethics. To overcome both of these concerns, the participants suggested that their research team have final say over any secondary analyses conducted by external researchers.

### Factor 3: balancing opportunities for public good against inappropriate uses of data

One of the most consistent messages from participants was that their decision to continue participating in the lifecourse research was driven by a desire to create public good. At the same time participants raised concerns about unacceptable reasons for external researchers to access aspects of data. Participants indicated that they would be agreeable to sharing their own data where this led to improved outcomes for individuals and communities:

“say I have a condition and somebody born has that condition and you share my information with the Ministry of Health to help that person, I would be happy about that. So more on a human level than on another level.” – P16

While the potential for data-sharing to create public good was clear to participants, they also found data-sharing hard to reconcile with the data being used for purposes outside the stated original intention. The process of gaining informed consent and specification about who can access data was queried by some participants:

“there is certainly an obvious line of argument that people have consented to give their data to the study […] they haven’t consented to give data to a research group in Arkansas or Delhi” – P1

Data-sharing was deemed unacceptable by most participants if it could be used outside the purpose of creating public good. Inappropriate outcomes that participants referred to included data being shared in a way that meant the research team had no control over future analyses, which may not always be for the public good. In addition, participants argued that data-sharing would not be aligned with public good if external researchers or funders are profiting financially from shared data. Some participants raised the issue as to whether the New Zealand government was an appropriate recipient of data, although some acknowledged that the government’s public funding meant that benefits for local communities were possible. However, several participants expressed that it was unclear why the government would be requesting data. Some Māori participants emphasised that negative experiences, both within and outside of research contexts, meant that they were reluctant for their data to be shared with government agencies. It is apparent that for these participants there are issues of trust based on past experiences:

“what needs to be clear is what are the benefits of making things available and who benefits from it? [...] if it is that it is a publishing house benefits at the financial level or a government department benefits in terms of its reputation or standing or how it appears in terms of its ability to discharge a changing set of expectations that a minister or the public might have of it, I don’t think they’re such strong drivers really” – P1

As conversations about data-sharing progressed, participants generally concluded that data-sharing would be deemed appropriate if two conditions were met: (a) if the explicit purpose of sharing data were to create public good; and (b) if the research team made the decision about whether the data should be shared:

“I sort of say well look those that then have control of that data, which is at the moment essentially yourselves, if you see ultimately the public benefit in having wider access to that data for studies, whatever, then I think in a way I’m putting my trust in you that you will make a good decision on that because the public benefit that you can perceive of someone having access to this data outweighs the relatively low risk of someone finding out information directly attributable to me.” – P10

## Discussion

The overall aim of the present study was to explore the perspectives on data-sharing of participants in the Dunedin Study birth cohort as an example of a long-running lifecourse study. The qualitative approach applied in this exploratory study allowed us to build an understanding of participant perspectives on data-sharing in relation to their ongoing participation in this research and also gain insight into the concerns that participants in this research have about data-sharing. The resulting grounded theory provides a novel preliminary model about why aspects of data-sharing might be seen as inappropriate for lifecourse research, as depicted in Fig. 1. The findings raise important considerations about how research participants’ perspectives should be prioritised especially as they have been surprisingly absent in discussions on data-sharing [3].

The central finding of this study is that lifecourse research participants take a critical approach to data-sharing requirements and therefore any implementation of data-sharing should require considerable explanation and more input than answering a yes/no question on each individual’s consent form. Participants’ willingness for their data to be shared was shaped by: (1) who will receive the data; (2) whether anonymity will remain once data are shared; (3) the sensitivity of specific forms of data, including data related to mental health, crime, or relationships, or Māori data; and ultimately, (4) the view that individual agreement to data-sharing should not override the best interests of the most vulnerable members of the cohort and the wider cohort in any study (Fig. 1). Furthermore, participants felt that researchers who collect the original data should have a say about which, if any, external researchers receive access to shared data, and how. While participants saw some merit in the argument that data being used outside its original purpose can generate more public good, this only extended to situations that would not compromise participants’ confidentiality and would retain the quality of research.

Specific issues related to Māori data that were raised by Māori participants reflect Indigenous data sovereignty principles and calls for Māori data to be subject to Māori governance [21, 37, 38]. Our finding that data are seen as having varying sensitivity echoes previous research showing that data about stigmatised issues (e.g., mental health data) are seen as less appropriate for sharing [12]. Māori views on data belonging to the collective and that some data (e.g., biological samples) have a cultural significance that results in certain restrictions being imposed on their use aligns with wider research that challenges hegemonic notions of data ownership [37, 38]. The finding that the willingness to share data was influenced by who will receive those data is consistent with past research suggesting that research participants are most likely to want their data shared with medical doctors, and least likely to want the recipient to be researchers for private companies [15]. Past research has revealed concerns about research data being linked to national databases such as healthcare data due to possible misuse of data [39]. The present study extends insights into these concerns in the context of Aotearoa/New Zealand by demonstrating apprehension about other researchers being able to access research data in addition to concerns about government agencies accessing research data. In contrast, our findings highlight the importance of the relationship between researchers and the research participants in a given study: if participants’ trust is established and maintained then the original researchers are trusted with decisions about data-sharing.

Our findings suggest that there is a requirement for increased information and transparency about data-sharing. These findings expand on past research that has shown research participants have varied understandings of common scientific processes, which in turn highlights the need for comprehensive informed consent processes [40]. A systematic review noted that, while research participants are generally aware of possible risks associated with taking part in research, they are less likely to be able to identify specific risks arising from participating [41]. In addition, past research on data-sharing has shown that participants who gave their blanket consent to all future uses of their data on signed consent forms were less likely to do so when verbally asked to give consent under the same conditions [11]. Data-sharing has the potential to become another misunderstood component of research, and our findings add to past findings that unspecified future uses of data are likely to lead to breaches of the requirements of informed consent [42]. The need for clarification of informed consent processes relating to data-sharing is particularly pressing given increasing global shifts to make data-sharing mandatory in order to receive funding from public sources such as the US National Institutes of Health [4] or to publish in a growing number of journals. Future research could consider how these findings relate to existing and new models of informed consent and could expand to other research designs beyond long-term lifecourse studies.

The interviews conducted in this study were led by the Director of the Dunedin Study rather than the primary analyst. This decision was made because the interviewer had existing rapport with the participants, and this is in keeping with best practice in semi-structured interviews, which rely on the rapport between the research participant and the interviewer [31]. At the same time, future research with external parties might provide different insights into participants’ experiences of lifecourse research that they might not be willing to explain to a researcher they will continue to interact with. In addition, the analysis was led by the first author as part of a Master’s degree and then as a staff member. No conflicts of interest were experienced within these arrangements and particular consideration was given to what could be concluded from interviews conducted by another member of the team. This enriched the analysis by requiring considerations of aspects of communication that might be taken for granted by someone present in the interviewing. Future research could explore alternative ways of gathering data about perspectives on data-sharing, potentially including more anonymised methods such as online surveys to reduce socially desirable responding or more open methods such as focus groups that provide insight by hearing discussion among participants and other stakeholders.

The sample was appropriately diverse based on purposive efforts to stratify based on gender, education, and ethnicity, although complete balance was not achieved. Future research should focus on particular demographics to ensure representation and would ideally involve interviewing all participants in the cohort. Although the study involved proactively recruiting Māori participants to ensure Māori voices were included, the study was not specifically focused on Māori views of data-sharing. A kaupapa Māori methodology would be a useful way to extend the findings presented here by conducting a study specific to the perspectives of Māori participants in lifecourse research that is led by Māori, with Māori, and for Māori [18].

While not an inherent limitation of the research itself, the changing macro-context of the global COVID-19 pandemic has meant that considerations of data storage and sharing have become more prevalent in daily life. In Aotearoa/New Zealand, Bluetooth location tracking, data-sharing between government departments, and COVID-19 tracing mobile applications have become part of the public discourse around nationwide management of the pandemic. Given this changing global context, participants’ perspectives and concerns about data storage, safety, and sharing may have changed, even if only relating to the data collected for COVID-19 tracing purposes. The periodic nature of the interview phases over several years worked to our advantage here, as approximately half of the participants were interviewed after the COVID-19 pandemic had begun. While some participants interviewed after the onset of the pandemic discussed how the pandemic had made them more aware of the types of data that were recorded for public health purposes, those scenarios were considered to be different from the collection of data for the Dunedin Study.

## Conclusions

Overall, this study provides novel and in-depth insights into research participants’ perspectives about data-sharing in the context of a long-running lifecourse study with implications for informed consent processes in such studies and potential impact on retention of participants in order to provide valuable long-term sources of knowledge about human health and development. The findings also inform researchers as well as ethics committees, journal editors, research funders, and government policymakers about balancing benefits of data-sharing against the need to inform and seek permission from participants in lifecourse research. However, we acknowledge that these arguments do not necessarily apply to other research designs, such as one-off surveys, and have less relevance for research projects that were established from the outset with the explicit intention of data-sharing, such as the UK Biobank [43].

The main implication of the present study is that informed consent processes relating to data-sharing need to be enhanced universally and be tailored to the specific community or population that is being studied. Given researchers’ roles in implementing stipulations about data-sharing, considerations about the possible sensitivity of data, who the data recipients are, consent models, and Indigenous data sovereignty principles should be considered by researchers in the planning stages of a prospective study. Another implication of the present findings is that consultation with research participants about data-sharing plans should be a mandatory component of the scientific research process. Further to this, consultation needs to cover present and possible future outcomes of data-sharing in lifecourse research and should be conducted verbally to ensure full understanding is achieved. Requiring participants to sign blanket agreements to all forms of data-sharing is problematic, and more responsive models such as dynamic or tiered consent are promising ways of overcoming concerns, but with implications for additional discussions with participants [40]. Future research with participants of lifecourse research could explore what models of informed consent meet the specific ongoing needs of participants and how this relates to feelings about uses of their own data and the data of whole cohorts that may be part of their identity as long-term study members.

Research participants have been a relatively overlooked stakeholder group in research and discussions on data-sharing in long-term lifecourse research, and this is surprising given that unplanned data-sharing has the potential to cause the most harm to participants out of all parties involved in this kind of research. Our findings demonstrate why these concerns are particularly relevant to lifecourse research given the ongoing and often life-long involvement of participants. Our study provides a model of the concerns and recommendations of participants that can inform policy and ethical practice. Transparency and rigour with how data-sharing is planned, explained, and implemented is urgently needed in lifecourse research.

## Data Availability

The dataset generated and analysed in the current study are not publicly available due to the confidentiality of participants, who are part of an ongoing lifecourse study. Any enquiries about the data can be addressed to the corresponding author.
